# The Educational Needs of Family of Patients Discharged from the Intensive Care Units: The Viewpoints of Nurses and the Patients' Families

**DOI:** 10.1155/2021/9956023

**Published:** 2021-04-30

**Authors:** Asma Hajalizadeh, Mehdi Ahmadinejad, Mahlagha Dehghan, Mansoor Arab

**Affiliations:** ^1^Nursing and Midwifery School, Kerman University of Medical Sciences, Kerman, Iran; ^2^Fellow of Critical Care, Anesthesiology and Critical Medicine, Kerman University of Medical Sciences, Kerman, Iran; ^3^Nursing Research Center, Kerman University of Medical Sciences, Kerman, Iran; ^4^Nursing and Midwifery School, Bam University of Medical Sciences, Bam, Iran

## Abstract

**Introduction:**

Thousands of patients are admitted to the intensive care units annually, which are stressful for patients and their families. The discharged patients and their families face different challenges in the caring process of the patients.

**Objectives:**

This study aimed to determine the educational needs of the families of patients discharged directly home from the postintensive care units and to compare the views of families and nurses about these needs.

**Method:**

This was a cross-sectional study. One hundred forty nurses and 140 family members of the patients discharged from intensive care units participated in the survey by convenience sampling method. A questionnaire of sociodemographic information and a researcher-made questionnaire on the educational needs of the family of patients discharged from the postintensive care units were used for data collection.

**Results:**

The mean total score of the educational needs of the patients' families was 31.81 and 35.33 from views of families and nurses, respectively. Nurses significantly estimated the educational needs of families more than what they did (*P* < 0.001). The families and nurses reported the educational needs of self-care as well as nutrition and medicine at the highest level, respectively. Both groups reported the educational needs of defecation at the lowest level. Nurses estimated higher educational needs in all dimensions, except for the patient's mental health and family self-care than families (*P* < 0.001).

**Conclusion:**

According to the present study, the educational needs were high from the views of nurses and families. Family need assessment is essential in designing and applying instructional interventions. Given the high level of family needs, implementing educational and practical interventions is necessary to enhance their skills.

## 1. Introduction

Thousands of patients are admitted to the intensive care units every year. Families believe that hospitalization of patients in the intensive care unit is the saddest event of their lives [1]. In this crisis, they will encounter some difficult realities such as the new environment, the unknown consequences, or even the death of their patient [2]. Since most patients in this unit are not able to decide on their treatment, families are responsible for decision-making and support for their patients, which are a huge responsibility. Therefore, attention to families is one of the essential components of the treatment process, which has somehow been neglected in the treatment system [2, 3].

Many discharged patients, depending on the physical and psychological injuries, need special care, which must be met by a trained person with full support of the family due to economic difficulties [4, 5]. The lack of familiarity and awareness of families about the type and method of proper care causes many problems and increases the patients' readmitting in intensive care units. Factors causing such problems include the lack of educational, regulatory, professional systems, and noncontinuous care. Therefore, comprehensive postdischarge training of caregivers will save costs and prevent readmitting of patients in hospitals [6, 7].

Nurses focus primarily on the needs of patients and often forget family needs. Nurses, like those in close contact with the patient, are ideal individuals to help family members meet the needs and deal with stressful situations [3]. Scientific family need assessment is the first step in preventing the inattention to patient and family's care that occurs in many cases unwantedly [7].

The high need of caregivers for training and educational deficits, such as intensive training during discharge, verbal training, inadequate training, and training in improper time, has led caregivers to seek instruction from professional and nonprofessional sources [4]. Therefore, the role of nurses, as professional staff, is highlighted. As a result, the nurse must first examine the patient and his or her family for training and prioritize, design, implement, and assess their educational needs, taking into account the level of literacy, culture, facilities, hospitalization time, and the status and ability of patient during discharge [8]. Training requires the accuracy of personnel and enough information because satisfaction with training is achieved [9] when there are an agreement and convergence between the educational expectations of patients and their families and the implemented training program [10, 11].

A study by Pagani et al. [12] found that one of the needs of caregivers of patients with a low level of consciousness or vegetative state was the secure communication with physicians and healthcare staff to receive information about complications and problems [12]. Buchini et al. in their study have recognized the lack of knowledge of vegetative care as one of the most common barriers to care provision [13]. On the other hand, the viewpoints of nurses and caregivers about the importance of caring needs can lead to a misconception of the needs of caregivers and, consequently, inappropriate care provision. The results of the studies showed that the treatment staff did not perceive the needs of the patients and the family properly [14, 15]. Therefore, it is imperative that nurses consider the views of patients and their families for educational needs.

A literature review showed that limited studies addressed the educational needs of the family of patients discharged from the medical wards [16, 17] and intensive care units [4]. Since education and caring for the family of the patients is one of the nurses' essential duties, awareness of their educational needs will enable nurses to provide appropriate care for patients and their relatives [3, 9, 11]. Providing nursing care based on family needs increases their satisfaction [10, 11]. Thus, the role of a nurse is a holistic one that should be both patient- and family-centered. Therefore, considering the importance of the role of the family in the postdischarge period of the patient from the intensive care unit, this study aimed to assess the educational needs of the families of patients discharged directly home from the intensive care units and compare the views of families and nurses about these needs in southeastern Iran.

## 2. Methods

### 2.1. Study Design and Setting

This is a cross-sectional study. The research setting was the intensive care units of two educational hospitals in Kerman, southeastern Iran. These centers provide specialty and subspecialty services for various patients in southeastern Iran. In the study setting, ICU patients with high care demands are discharged directly home after staying a few days in the Post-ICU Department. There are some facilities available such as home care nurses; however, using these facilities depends on the family willing and economic status. In addition, there are some governmental and nongovernmental organizations which provide some equipment including portable mechanical ventilator, hospital bed with wavy mattress, and catheters, free of charge. However, the families are 100% responsible for the patient's care.

### 2.2. Sample Size and Sampling

The sample size with a confidence interval of 95% was estimated to be 123 individuals in each group using a pilot study on 15 nurses and patients' families (_*µ*1_ _=_ 3.9, _*µ*2_ _=_ 3.54, *S*1 = 0.6, and *S*2 = 0.94). Regarding the probability of dropout, 140 samples were considered in each group. The convenience sampling method was used for sampling.

### 2.3. Instrument

The instrument was a researcher-made questionnaire based on background information and educational needs. The patient's family background information includes age, sex, marital status, educational level, occupation, monthly income of the family, and relationship with the patient. The patient's background information includes age, sex, marital status, educational level, insurance coverage, patient diagnosis, hospitalization time, the time elapsed from discharging, the status of the patient when discharging from the ICU, and the patient's current condition. Nurses' background information includes age, gender, marital status, educational level, work experience, work experience in the ICU, position, working hours per month in the ICU, type of shift, and type of employment.

The questionnaire of educational needs of patients discharged from intensive care units was designed by interviewing family members of discharged patients, experienced nurses, physicians working in the intensive care unit, and personnel working in rehab centers as well as using a literature review. The questionnaire consisted of 67 items and eight dimensions. The dimensions of the questionnaire include (1) public health (9 items about hand hygiene, patient's oral care, eye care, skin care, bed sore prevention, care for bed sore, bath in bed, limb physiotherapy, and care of patients in winter), (2) airway care (15 items about the tracheostomy care and suctioning, use of home ventilator and oxygen container/oxygen maker, use and disinfection of oxygen mask, nasal cannula, t-piece, Ambo Bag, chest physiotherapy, and knowledge of lung infection symptoms), (3) urinary care (8 items about care of internal and external urinary catheter, urine volume measurement, and urinary tract infection symptoms), (4) defecation care (6 items about the frequency of bowel movements and stool color, prevention and control of constipation, prevention and control of diarrhea, use of bedpan for the patient, and removal of the ostomy pouching system and its reinstallation), (5) nutrition and medicine (9 items about preparation and frequency of the patient feeding, feeding through the nasogastric tube or gastrostomy, NGT care, drugs administration to the patient through the nasogastric tube, subcutaneous/intramuscular drugs administration, knowledge of drug side effects), (6) mental health of the patient (3 items about control of the patient's mental tensions and stresses, depression, and psychological issues), (7) essential information (11 items about control of the patient's level of consciousness, vital signs, fever, pain relief, blood clots prevention, care for the patient during seizure, basic cardiopulmonary resuscitation, removal of tracheostomy tube and its reinsertion, and information about the rehabilitation centers for ICUs), and (8) self-care (6 items about control of stress and mental tensions caused by caring for the patient, taking care of his/her physical health, psychological adaptation to the patient's condition while caring for him/her, control of anger in dealing with the patient's condition, and managing and planning for patient care). The response to the questionnaire was based on the level of family needs from no needs = 0 to very high needs = 5. The mean scores allocated to each item were used to calculate the educational needs of each dimension, so the minimum score for each dimension was 0, and the maximum score was 5. The sum scores of all dimensions were calculated to assess the total score of the questionnaire. Therefore, the total score ranged from 0 to 40, and the higher the score, the higher the educational needs. Also, scores from 0 to 13.34 were considered as low, from 13.35 to 26.67 as moderate, and 26.68 to 40 as high educational needs. Sixteen faculty members of Razi Faculty of Nursing and Midwifery and specialists of the intensive care unit assessed the questionnaire for content validity. The content validity index was 0.99. The questionnaire was given to 30 target people (15 nurses and 15 family members of the patient) to determine the reliability, and Cronbach's alpha coefficient was 0.91. The full version of the questionnaire was published elsewhere [18].

### 2.4. Data Collection and Analysis

The researcher started the sampling after obtaining the code of ethics and permission from the research setting. The researchers extracted patients' families contact information from the medical records of patients who have been discharged from the intensive care units since 2016. After contacting eligible families, an appointment was arranged at their living place, at the rehab center, and, in some cases, in nursing care clinics to fill out the questionnaires. Initially, a description of the research process, its goals, and the confidentiality of the information was provided for the research units. In case the primary caregiver had not had sufficient literacy, the questionnaire would have been completed through the interview. Also, the researcher coworker was available for literate families in the case of having any question. Because the educational needs of the family may differ depending on the patient's condition after discharge and this will affect the nurses' viewpoints, nurse samples were matched with the family samples according to patients' conditions. The researcher referred to teaching hospitals during different shift works (morning, evening, and night) and provided the nurses with questionnaires. Descriptive statistics (frequency, percentage, mean, and standard deviation) were used to describe the characteristics of the samples. An independent *t*-test was used to compare the family educational needs of caring for the patients discharged from the intensive care units from nurses and families' viewpoints. Data analysis was done using SPSS version 21.

### 2.5. Ethical Considerations

The XXX ethical committee approved the research (IR.KMU.REC.1396.1699). Caregivers were also assured that the collected information would be confidential and used only for research purpose. Written informed consent was taken from caregivers of patients and nurses.

## 3. Results

The mean age of nurses was 35.33 ± 8.79 years, the mean work experience was 5.50 ± 2.75 years, the mean work experience in the ICU was 5.11 ± 2.75 years, and the mean working hours per month were 126.20 ± 39.77. 84.3% of nurses were female, and 15.7% were male. 67.1% of nurses were married, and 31.4% were single. The majority of nurses had a B.S. degree.

The mean age of the family members of the patients was 40.86 ± 11.4 years. 70.7% of the patients' families were female, and 29.3% were male. 72.9% were married, and 20% were single. 25.7% of the caregivers had an academic degree. Most caregivers were housewives. 41.4% had an income below one million tomans (Iran currency).

The mean age of the patient was 40.02 ± 17.38 years, and the time elapsed from the patient's discharge from the intensive care unit was 129.72 ± 192.74 days. 54.3% of the patients were female, and 64.3% were married. 22.1% of patients had an academic degree. All patients were insured. 45.7% of the patients were hospitalized in the ICUs for neurosurgeries, 17.8% for orthopedic surgeries, 7.1% for heart surgeries, 12.9% for other general surgeries, 8.6% for neurology disease, and 7.9% for other medical diseases. 97% of patients were discharged from ICU with some degree of unconsciousness, while 76% of them were in coma status. 27.9% of them needed mechanical ventilation at home. 56.4% of them were discharged from ICU with a tracheostomy. 71.4% of them had bedsores at the time of discharge. 83.6% of them needed enteral nutrition. 77.1% of them needed oxygen support at home. 87.1% of them needed suctioning at home. 92.1% of them needed help for changing position. 94.3% of them needed internal or external urinary catheterization. 47.1% had a gastrointestinal ostomy. 67.9% of them needed dressing changing, and 81.4% of them needed intravenous/intramuscular drugs.

The mean total score for the educational needs of the family of patients was 31.18 from the viewpoint of families and 35.33 from the nurses' point of view. There was a significant difference between nurses and families' viewpoints in the mean score of educational needs so that these educational needs were higher from the nurses' point of view. Among the different dimensions of educational needs, self-care (4.89) was the highest educational need, and defecation (3.23) was the lowest educational need from the viewpoint of families. The nutrition and medication (4.7) were the highest educational needs, and defecation (3.97) was the lowest educational need from nurses' point of view. Nurses rated higher educational needs in all dimensions, except for the patient's mental health and self-care dimensions than families. There was a significant difference between nurses and families in all dimensions (Table 1).

12.9% of families considered the severity of the needs at a moderate level, and 87.1% considered it at a high level, while all nurses reported a high degree of needs. There was a significant difference between families and nurses in the severity of needs (Table 2).

Also, there was a statistically reverse significant association between families' age and informational needs so that the older the age, the lower the informational needs, and vice versa (*r* = −0.2; *P*=0.02). There was no significant association between the families' informational needs and other demographic characteristics (*P* > 0.05). Also, there was no significant association between the families' informational needs and sociodemographic characteristics of the nurses (*P* > 0.05).

## 4. Discussion

The results of the study showed that, in general, educational needs and most dimensions in the family of patients discharged from the intensive care units were very high from the views of families and nurses working in these units. In a study in Iran, the caregivers of the patient in a vegetarian state experienced many challenges in the care of their patients and required some education but received partial training at the time of discharge. They had to seek training in various sources after discharge [4].

In the present study, the educational needs of self-care, patient mental care, and essential information were at a high level from the viewpoint of families (score above 4 out of 5), and other educational needs were above the average (score above 3.23 out of 5). From the nurses' point of view, the severity of all the educational needs of the family of patients (higher than 4 out of 5) was significant except for defecation (3.97 out of 5). Unfortunately, limited studies have addressed the educational needs of the family of the patients discharged from the intensive care units, and most studies have addressed the needs of the discharged patients. The results of the study by Bassampour et al. [17] showed that the majority of patients and their families had moderate to high educational needs about surgical wound care, had moderate educational needs about activities, and had low to moderate educational needs about rest, drug use (general educational needs), nutrition, and other needs. The majority of patients and their families had moderate educational needs in all aspects one month after discharge [19]. The results of this study were consistent with those of the present study. The results of the study by Alaviani et al. [16] showed that patients discharged from the medical ward needed to be cared for the intubation (100%), wound (100%), the activity and mobility (92%), and diet (60%). Patients discharged from the surgical wards needed to be cared for medication use (54.7%), diet (68%), physical activity (94.7%), the plastered limb, incubation, and wound (100%). In line with the present study, the level of needs was high [20]. Kerzman reported in his research that patients' knowledge was low, and 40% of the patients were not provided with training [7].

In this study, different aspects of the educational needs of the family, that is, public health, airway care, urinary care, defecation care, nutrition and medicine, and essential information, were considered higher in nurses' viewpoints compared to the families, while families estimated higher educational needs for the mental health of patients and self-care than nurses. The differences between families and nurses' viewpoints were also clinically important, as it is obvious regarding the effect sizes (range from 0.79 to 1.77 for different aspects of family informational needs). Therefore, although both groups took into account the needs at a high level, there was a difference between the two groups. In other words, the importance and priority of needs were different from the viewpoint of the family of patients and nurses. The results of studies have shown that the healthcare staff has not adequately perceived the needs of the family, and this can lead to inappropriate care provision [7, 21]. In line with the results of the present study, the results of the survey of Mohammad Pour and Dehghan Naieri showed that the provided training was enough from the patient's viewpoint (65.7%), but only 56.8% of them had adequate knowledge [22]. However, nurses reported some needs of the family at a lower level than families, which may be due to the research setting. The focus of nurses in our hospitals is on the physical needs of patients, and less attention is paid to the mental needs of patients and their families. Cameron et al. [23] showed that 67% of caregivers of patients discharged from the intensive care unit reported high depression at the beginning, and 43% reported it one year after discharge [24]. Critical illness and postdischarge problems of patients in ICUs have long-term complications for caregivers of these patients, including psychological factors such as depression, anxiety, posttraumatic stress syndrome, panic, and aggression, and thus reduce the quality of life of caregivers [25]. Therefore, although families and nurses reported the educational needs of the patient's family at a high level, according to the results of various studies, nurses did not provide proper education regarding the needs of patients and their families. For example, Hegney et al. reported that less than 50% of nurses trained patients about drug therapy [24]. Also, Khezrloo et al. [25] showed that the educational role of nurses was 42% and 66.9% of them had an undesirable educational role, 23.4% had a relatively desirable role, and only 9.7% had a desirable role [25]. Therefore, it is necessary to pay particular attention to the education of the families of patients, especially the family of patients discharged from intensive care units. Family-centered education can be provided with proper need assessment of these families.

One of the limitations of this study was to use a questionnaire to examine the educational needs of families. In other words, some people may refuse to provide real responses and give an unrealistic answer. According to the sampling method and the research setting, the results of the study should be generalized with caution. In addition, the cross-sectional design of the present study does not allow conclusions on cause-and-effect relationships. Other limitations of this project can be individual characteristics, psychological characteristics, and cultural, social, and living differences of the samples that influenced the answers to the research questions and they were out of control of the researcher.

## 5. Conclusion

The results of this study showed that, from the viewpoints of the families of patients discharged from the intensive care units and nurses working in these units, families of these patients have high educational needs in different areas of caring for the patients discharged from the ICU. In addition, informational needs about self-care and mental health of the patient had high priority from the families' viewpoints. Many patients who are discharged from ICUs still need care for reasons such as reduced consciousness, mechanical ventilation, and enteral nutrition. Unfortunately, in Iran, the focus of facilities and resources is limited to two primary and secondary prevention levels, and rehabilitation and follow-up care, such as home care, have been less considered. Therefore, according to the results of this study, it is suggested that educational programs be designed and implemented for the family of these patients. Also, nurses should design flexible educational programs based on the level of family perceptions. Further research is needed to evaluate the family's performance in caring for a patient discharged from the intensive care units as well as the function of ICU nurses in family education.

## 6. Clinical Significances

The majority of the families of patients discharged from intensive care units had high educational needs. The educational needs of self-care, patient mental care, and essential information were at a high level from the viewpoint of families. The educational needs of airway care, public health, urinary care, deification care, nutrition, and medication were at a moderate level from the viewpoint of families. Nurses in intensive care units have an important role in prevention, early diagnosis, and taking care of patients and their families in ICUs. Nurses believed that the patients' family educational needs are at a high level in almost all dimensions of caring patients discharged from intensive care units. Healthcare providers, especially nurses, should be aware of the educational needs of family members of the patients discharged from ICUs to provide better care. Therefore, it is recommended that these topics be included in the healthcare provider's curriculum and nurses with greater academic ability, higher perception, and more top skills be applied in intensive care units.

## Figures and Tables

**Table 1:** Comparing mean scores of educational needs of the families of patients discharged from ICU and their dimensions from nurses and families' points of view.

Variable	Patients' families		Nurses		Independent *t*-test	Effect size	*P* value
	Mean	SD	Mean	SD			
Public health	3.70	0.57	4.50	0.29	−11.25	1.77	<0.001
Airway care	3.86	0.70	4.63	0.29	−10.79	1.44	<0.001
Urinary care	3.51	0.85	4.31	0.44	−8.27	1.18	<0.001
Defecation care	3.23	1.11	3.97	0.71	−5.39	0.79	<0.001
Nutrition and medication	3.83	0.69	4.70	0.24	−11.39	1.68	<0.001
Patient's mental health	4.76	0.29	4.19	0.70	−6.76	1.06	<0.001
Essential information	4.25	0.46	4.63	0.29	−6.92	0.99	<0.001
Self-care	4.89	0.19	4.29	0.72	−8.47	1.14	<0.001
Total score	31.18	3.97	35.33	2.6	−8.47	1.24	<0.001

SD: Standard Deviation.

**Table 2:** Comparison of the severity of the educational needs of the patients discharged from the intensive care units from the viewpoint of nurses and families.

Educational needs of the family	Group					
	Families		Nurses		Exact Fisher test	*P* value
	Frequency	Percentage	Frequency	Percentage		
Moderate	18	12.9	0	0	19.24	<0.001
High	122	87.1	140	100		

## Data Availability

The datasets used for the current study are available from the corresponding author upon request.
